# Bone Mineral Density as a Predictor of Atherogenic Indexes of Cardiovascular Disease, Especially in Nonobese Adults

**DOI:** 10.1155/2019/1045098

**Published:** 2019-09-04

**Authors:** Tzyy-Ling Chuang, Jiunn-Wen Lin, Yuh-Feng Wang

**Affiliations:** ^1^Department of Nuclear Medicine, Dalin Tzu Chi Hospital, Buddhist Tzu Chi Medical Foundation, Chiayi, Taiwan; ^2^School of Medicine, Tzu Chi University, Hualien, Taiwan; ^3^Department of Cardiology, Dalin Tzu Chi Hospital, Buddhist Tzu Chi Medical Foundation, Chiayi, Taiwan

## Abstract

**Purpose:**

This study is aimed at determining whether bone mineral density (BMD) values are related to atherogenic indexes (AIs) and could predict the risk of cardiovascular disease (CVD) in southern Taiwanese adults.

**Methods:**

Medical records of 3249 adults who underwent health examinations between June 2014 and February 2018 at a regional hospital in southern Taiwan were reviewed. Data collected included health history, anthropomorphic characteristics, exercise habits, diets (vegetarian or nonvegetarian), clinical laboratory results (lipid profile, systemic blood pressure (SBP), glucose level, creatinine (Cre) level, and hemoglobin (Hb) level), and bone mineral density (BMD), which were used to identify the associations of these parameters, especially BMD, with lipid profile and calculated AIs through simple and multiple linear regressions.

**Results:**

The mean age of the patients was 58.0 years, and 71.4% were male. Body mass index (BMI), SBP, glucose level, Cre level, Hb level, and all BMD values were positively correlated with triglyceride (TG) level and AIs and were negatively correlated with high-density lipoprotein cholesterol (HDL-C) level. The significant positive correlations of BMD at all the measured sites with AIs remained after adjusting for age, sex, SBP, glucose level, Cre level, Hb level, smoking, exercise habits, and vegetarian state. The expanded adjusting model for TG/HDL-C remained significant at all the BMD measured sites in nonobese men, at bilateral femoral neck and total hips in nonobese women, and at the bilateral total hips in obese women.

**Conclusions:**

AIs are predictive markers for CVD, and BMD values are predictors of AIs, especially the novel AI, i.e., TG/HDL-C ratio, in nonobese adult men and women after dividing the patients into subgroups to eliminate the effect of BMI as a confounding factor. Thus, BMD values could predict AIs of CVD, especially in nonobese adults.

## 1. Introduction

Family history, age, smoking, hypertension, diabetes mellitus, low physical activity, and obesity are risk factors for coronary artery disease (CAD) [1]. Traditional lipid profile includes triglyceride (TG), total cholesterol (TCH), and low-density lipoprotein cholesterol (LDL-C), which are all atherogenic, and high-density lipoprotein cholesterol (HDL-C), which is cardioprotective [2–4]. Atherogenic indexes (AIs) are ratios that have atherogenic particles in the numerator and HDL-C in the denominator [5]. The ratios of TCH/HDL-C, LDL-C/HDL-C, and TG/HDL-C (a recent most novel index) could be predictors of CAD risk and extent [1, 5, 6]. Moreover, TCH/HDL-C and LDL-C/HDL-C ratios are useful and simple indexes of ischemic heart disease and cardiovascular disease (CVD) [1, 7, 8]. TG/HDL-C value is found to be a good marker for cardiometabolic risk ratio, and Log(TG/HDL-C) may be a strong marker for predicting the risk of CAD [8, 9].

Previous studies suggested that atherosclerosis and osteoporosis are linked by common risk factors and pathomechanisms [10–12]. Atherosclerosis could include calcified, noncalcified, or mixed lesions. Our previous study suggested that bone microarchitecture remodeling becomes more active when early coronary artery calcification (CAC) occurs [13]. A prediction model based on hypertension, hyperlipidemia, TG, and L-spine *T*-score explained 73.2% of the variance of CAC [14]. Evaluating the correlation between CVD and bone mineral density (BMD) should take their respective factors into consideration. For example, age and menopause act on both in the same direction, whereas body mass index (BMI) acts on them in different directions [15].

The relationship of BMD and lipid profile, especially AIs, remains to be clearly established. HDL-C is reported to be inversely related to BMD [15–17]; however, some studies reported no [18] or positive [19] correlation between HDL-C and BMD. Results of other lipids showed inconsistency [18–23]. A previous study reported that TCH/HDL-C was positively correlated with both femoral neck BMD and total hip BMD [24]. This finding motivated us to conduct this study. Hence, our study is aimed at clarifying the relationship of BMD with AIs and at identifying whether BMD could be an indirect or direct predictor of CVD.

## 2. Materials and Methods

### 2.1. Subjects

This retrospective study included adults who underwent health examinations between June 2014 and February 2018 at the preventive medical center of a regional teaching hospital in southern Taiwan. Subjects who previously underwent internal fixation or total hip replacement at sites where BMD was measured were excluded. This study was approved by the ethics committee of our institution, who waived the requirement for informed consent from each patient. Questionnaire records included (1) history of hypertension, diabetes mellitus, and hyperlipidemia; (2) history of smoking and drinking; (3) anthropomorphic characteristics (age, sex, and BMI); (4) exercise habit of greater or equal to 3 times a week; and (5) diet habit of vegetarian or nonvegetarian.

BMI was defined as a person's weight in kilograms divided by the square of the person's height in meters (kg/m^2^). We also divided the population into four subgroups according to sex and obesity status. Classification of nonobese (BMI ≤ 24.9) and obese (BMI ≥ 25.0) subjects was based on the values indicated by the World Health Organization.

### 2.2. Laboratory Data

The clinical laboratory findings obtained during health examinations included the following: TG, TCH, LDL-C, HDL-C, systolic blood pressure (SBP), glucose level before meal, creatinine (Cre) level, and hemoglobin (Hb) level. The three AIs in this study were TG/HDL-C, TCH/HDL-C, and LDL-C/HDL-C (atherogenic particles in the numerator and HDL-C in the denominator).

### 2.3. Bone Mineral Density

BMD was assessed by dual-energy X-ray absorptiometry (DXA) using a Discovery Wi DXA system (Hologic Inc.). The areas where BMD was measured included the lumbar spine, the bilateral femoral neck, and the total hip regions. The same densitometer was used for all the patients to ensure accurate comparisons.

### 2.4. Statistical Analysis

Results were expressed as mean ± standard deviation or number (percentage), as appropriate. Differences in means or frequencies were tested using the chi-square test or *t*-test, as appropriate. Simple linear regression analysis between the parameters and lipid profile and AIs was performed. Simple linear regression between the possible confounding parameters and BMD values was also analyzed. Multiple linear regression analysis was conducted using BMD values of each measured site as the independent variable and AIs as the dependent variables for three models adjusted for the following: (1) age and sex, (2) BMI, and (3) age, sex, SBP, glucose, Cre, Hb, smoking, exercise, and vegetarian. Further, multiple linear regression analysis of the novel AI (i.e., TG/HDL-C) and BMD values was performed for the subgroups after adjusting for age, SBP, glucose level, Cre level, Hb level, smoking, exercise, and vegetarian. All statistical analyses were performed using PASW Statistics 18 suite (SPSS Inc., Chicago, IL).

## 3. Results

### 3.1. Subject Characteristics

Medical records of 3249 adults were included. The demographic and clinical characteristics of the subjects are presented in Table 1. The study population was predominantly male (71.4%), with a mean age of 58.0 ± 11.2 years. Analysis of clinical characteristics indicated that 26.9% of the patients had hypertension, 9.6% had diabetes, and 6.9% had hyperlipidemia; moreover, 5.2% were smokers, 42.8% had the habit of exercise, and 42.8% were vegetarians. Significant differences between male and female subjects in smoking, exercise, or diet habits, BMI, SBP, Cre, Hb, TG, TCH, HDL-C, and three calculated AIs were found.

### 3.2. Simple Linear Regression

The lipid profile and AIs were the dependent variables. Age showed significant negative associations with all the lipid profile parameters, except HDL-C level, which showed no correlation. BMI, glucose level, and Hb level showed significant positive correlations with all the lipid profile parameters, except HDL-C level, which showed a negative association. SBP and Cre level were positively correlated with TG level and the three AIs and were inversely correlated with HDL-C level. Male gender and smoking habit were positively correlated with all lipid profile and AIs. Exercise showed negative correlation with atherogenic lipid profile and AIs. Vegetarian diet had negative relationship with TCH, LDL-C, HDL-C, TCH/HDL-C, and LDL-C/HDL-C (Table 2).

### 3.3. Bone Mineral Density

Mean lumbar spine BMD values on DXA were 0.988 ± 0.148 g/cm^2^ for men, 0.878 ± 0.146 g/cm^2^ for women, and 0.957 ± 0.156 g/cm^2^ for all patients. Significant differences in BMD values of the lumbar spine, bilateral femoral neck, and total hip regions between males and females were observed (Table 1).

Moreover, BMD of all the measured sites was significantly positively correlated with TG level and the three AIs and was negatively associated with HDL-C level. All BMD values, except the left femoral neck BMD value, were negatively correlated with TCH level. BMD values at all the sites had no statistically significant relationship with LDL-C levels (Table 2).

For all measured sites, elder age and vegetarian had lower BMDs; moreover, male gender, BMI, Cre, Hb, and smoking had positive effect with BMDs. SBP and glucose showed positive correlation with BMD values of lumbar spine and bilateral total hips. Exercise only had significant positive relationship with the left total hip BMD (Table 3).

### 3.4. Multiple Linear Regression

In the analysis of the association of BMD and AIs, multivariate linear regression showed that BMD values at all the regions were positively correlated with AIs in model 1 and model 3 (Table 4). However, in model 2 (adjusted for BMI), all BMD values had a positive correlation with TG/HDL-C ratio but had no relationship with LDL-C/HDL-C ratio. In addition, only the total hip BMD values were positively correlated with TCH/HDL-C ratio (Table 4).

### 3.5. Subgroup Characteristics

Of the 3249 subjects, 1998 were nonobese (women 656 and men 1342) and 1251 were obese (women 273 and men 978). Multiple regression analysis at sites of BMD and AI of TG/HDL-C ratio in subgroups dividing by sex and obesity after adjustment of age, SBP, glucose, Cre, Hb, smoking, habits of exercise, and vegetarian diet showed major significant positive correlations in all regions at male nonobese subgroup, in bilateral femoral neck and total hip regions at female nonobese subgroup, and in bilateral total hip regions of obese females (Table 5).

## 4. Discussion

Lipid abnormalities, such as elevated TCH and LDL-C levels or low HDL-C level, are related to atherosclerosis [5], and TCH/HDL-C and LDL-C/HDL-C ratios have been shown to be good predictors of CAD than lipid alone [1, 4, 6]. High TG-low HDL-C dyslipidemia, which is the consequence of abdominal obesity and insulin resistance, is commonly associated with an increased concentration of small, dense LDL particles, which are atherogenic [1]. Although some lipid variables are associated with CAD extent, the TG/HDL-C ratio showed the strongest association with CAD extent [5]. Moreover, a previous study of Chinese patients with type II diabetes with stable CAD demonstrated that elevated TG/HDL-C ratio could also be a useful predictor of future cardiovascular events [25].

Yamaguchi et al. and Poli et al. found an inverse association between BMD and serum LDL-C levels in postmenopausal women, but somewhat different in the BMD measured sites [18, 19]. However, Adami et al. reported a significant positive association of serum LDL-C levels with BMD in elderly female subjects [20]. Inconsistent findings of the relationship of TCH level with BMD were also reported; moreover, some studies showed no significant correlation [21, 22]. In a South Korean population-based study of subjects aged 19–80 years, TCH and LDL-C levels were inversely associated with BMD in both premenopausal (*N* = 375) and postmenopausal (*N* = 355) women [23]. In a 2018 meta-analysis of postmenopausal women, TCH and LDL-C levels were higher in the osteoporosis group than in the normal bone density group [26]. In our study, we showed inverse relationships of TCH level with BMD at some sites (L-spine, right femoral neck, and bilateral total hips); however, no relationship between LDL-C level and BMD was observed.

In a prospective cohort of adults with type 1 diabetes and low-to-normal TG levels, TG at baseline independently predicted CAC progression over 6 years [27]. A previous study also showed that TG levels have a significant positive correlation with BMD values at the trochanter site among postmenopausal Korean women and a negative correlation with lumbar BMD values in the premenopausal Korean women [23]. A study in Saudi Arabia showed that glucose and TG are positively associated with total hip BMD [24], which is consistent with the findings of a related study in Italy [20]. In our study, which involved Taiwanese adults, TG levels were shown to have a consistent significant positive correlation with BMD values at all the measured sites.

Furthermore, our study showed an inverse relationship between HDL-C level and BMD values at all the sites. In contrast, previous studies showed no [18], positive [19], or negative [15, 17] relationship in postmenopausal women. Moreover, a negative relationship was noted in healthy women and men after adjusting for weight, height, and fat mass in an Italian study [20]. HDL-C levels were not associated with BMD values at any of the sites in pre- and postmenopausal subjects in a study in Korea [23]. These differences could be attributed to different study populations, age distribution, race, statistical methods, and/or adjustments.

Alissa et al. reported that TCH/HDL-C ratio was positively correlated with both femoral neck BMD and total hip BMD in 180 postmenopausal women recruited from a catheterization laboratory [24]. Adami et al. also reported that LDL-C/HDL-C ratio in women showed significantly positive relationship with BMD values after adjusting for body weight, height, and fat mass [20]. A significant negative relationship was found between whole-body bone mineral content and HDL-C/LDL-C ratio in Chinese men and women [28]. In our study, we expanded the sample size to 3249 Taiwanese adults undergoing health exam, and BMD values at all the measured sites showed significant positive associations with all three AIs (TG/HDL-C, TCH/HDL-C, and LDL-C/HDL-C ratios) in nonadjusted and some adjusted conditions.

Log(TG/HDL-C) is a strong marker for the prediction of the risk of atherosclerosis, CAD, and CVD [7]; reflects the true relationship between protective and atherogenic lipoprotein; and is associated with the size of a pre- and antiatherogenic lipoprotein particles [29]. And it is significantly positively correlated with BMI [7], so as our AI markers by naive linear regression without log. Similar to another study of HDL-C and BMD [15], the significant correlation of the novel AI, i.e., TG/HDL-C, with BMD disappeared when the correlative variables of sex and BMI were introduced. Hence, we divided our population into subgroups according to sex and BMI (obesity or nonobesity). After adjusting for age, SBP, glucose level, Cre level, Hb level, smoking, exercise, and vegetarian state, BMD at nonobese adults, except lumbar spines of nonobese women, still showed a significant positive relationship with TG/HDL-C ratio and can predict it. This is the most concrete evidence to support the purpose of our study. Moreover, total hip BMD values also could predict TG/HDL-C in the female obese subgroups. The lack of significance of lumbar spine BMD of nonobese women could be explained by the difficulty in lumbar spine BMD measurement in elderly subjects and the possibility of obtaining higher values owing to degenerative changes, such as aortic calcifications or osteophytes, which could in turn result in a “no association” finding [23]. To determine the true current status and a possible altered physiological condition after treatment, models were adjusted for clinical laboratory data instead of comorbidities.

Factors influencing BMD include age, sex, BMI, smoking, race, glucocorticoids, hyperlipidemia, and diabetes [30, 31]. A strong positive correlation between Cre clearance or Cre and bone mass or BMD values exists [32]. Our study also showed significant linear associations between Cre and TG levels and between Cre and AIs and an inverse correlation between Cre and HDL-C levels. A previous study found partial positive associations between Hb level and BMD in men but negative associations in women [33], whereas another study showed no relationship [34]. Interestingly, our data showed a significant positive association of Hb level with lipid profile, except HDL-C level, which showed a significant negative relationship, and AIs. Despite inconsistent results in the literature on smoking and BMD [35], they often showed inverse relationship [36]. Our opposite result (positive relationship) is probably due to the small percentage of smoking and older age of the nonsmoking population (58.4 ± 0.2 vs. 50.8 ± 0.9 years, *p* < 0.001). Significant high atherogenic indices and proatherogenic lipids (TG, TCH, LDL-C, very LDL-C, and non-HDL-C) were observed in smokers compared to controls [37, 38]. Exercise benefits BMD in premenopausal women [39] and maintains BMD in postmenopausal women [40]. Regular exercise improves HDL-C and decreases TG, TCH, LDL-C, and VLDL [41]. Physical exercise improves not only lipid profile but also AIs [42, 43]. Long-term practitioners of vegan vegetarian were found to being classified as having osteopenia of the femoral neck [44]. Another study showed that the proportion of subjects with osteopenia or osteoporosis also appeared comparable between vegetarians and nonvegetarians of both sex [45]. Vegetarian diets effectively lower blood concentrations of TCH, LDL-C, HDL-C, and non-HDL-C, but vegetarian diets did not significantly affect blood TG concentrations [46]. After adjustment for age, sex, SBP, glucose level, Cre level, Hb level, smoking, exercise, and vegetarian state, BMD values at all the measured sites showed a significant positive correlation with the three AIs.

Bone turnover is not simply regulated by osteoblasts and osteoclasts that produce and resorb bone, respectively. Bone formation involves other more complex elements at the molecular level. Atherosclerosis and vascular calcification are complicated dynamic processes related to the regulation of oxidized lipids, leptin, bone matrix proteins (osteopontin, osteocalcin, and bone morphogenetic protein), calcification inhibitors (osteoprotegerin, matrix Gla protein, and fetuin-A), osteogene expression, and inflammatory cytokines (TNF-*α*, CRP, and CD40-CD154) [11, 13, 47–50]. Moreover, exogenous lipids have been implicated in the regulation of osteoblastic differentiation [51, 52], and the products of cholesterol biosynthetic pathway are important for the proper development of marrow stromal cells. Studies have found that hypercholesterolemia and the resulting deposition and oxidation of lipids in tissues result in atherosclerosis [53] and are related to osteoporotic bone loss [54]. Thus, atherosclerosis is related not only to lipids but also to bone biosynthetic factors.

The conflicting findings between our study and those in some previous studies on the association of low BMD with cardiovascular risk or events [24, 55, 56] could be due to the difference in the interaction of calcified and noncalcified atherosclerosis with lipid pathway and could be because our study sample was composed of mostly normal and asymptomatic adults undergoing a health examination. Trabecular bone score value positively predicted moderate CAC but showed no association with the high CAC group in a previous study [13]. Non-HDL-C, which is a marker of subclinical atherosclerosis, was more strongly associated with CAC than with all other conventional lipid values [57]. Noninvasive methods could determine the presence and extent of CAD, especially by CAC, HDL-C, and TG/HDL-C ratio assays [58]. The use of BMD as a predictor of AIs of CVD is a novel concept.

However, this is a retrospective study and has some limitation. First, we had no information of body fat percent or fat mass, which may be confounding factors on the association between the BMD and AIs. Second, we did not have the medication data either. Lipid-lowering medications, such as statins and fibrates, have some effects on lipid profiles. Especially, statins are thought to have significant effects on both lipid profiles and BMD. However, since the percentage of hyperlipidemia is 6.9% and the population size is 3249 subjects, the possible interference to our result could be eliminated.

## 5. Conclusions

AIs are significant predictors of CVD, and BMD values are predictors of AIs. Observation showed significant positive relationships of BMD with the three AIs and especially with the AI of TG/HDL-C in nonobese men (at all the sites), nonobese women (at the femoral neck and total hip), and obese women (at the total hip) after dividing the study subjects into subgroups and after further adjustments to eliminate the effect of BMI as a confounding factor. Thus, BMD values could predict the AIs of CVD, especially in nonobese adults.

## Figures and Tables

**Table 1:** Demographic and clinical characteristics of study participants.

*N*	All	Male	Female	*p* value
	3249	2320	929	
Age (range) (years)	58.0 ± 11.2 (16–90)	57.9 ± 11.4 (16–90)	58.4 ± 10.6 (20–88)	0.300
Smoking (%)	169 (5.2)	165 (7.1)	4 (0.4)	**<0.001**
Hypertension (%)	875 (26.9)	633 (27.3)	242 (26.0)	0.473
Diabetes mellitus (%)	311 (9.6)	229 (9.9)	82 (8.8)	0.361
Hyperlipidemia (%)	225 (6.9)	160 (6.9)	65 (7.0)	0.919
Exercise	1389 (42.8%)	1048 (45.2%)	341 (36.7%)	**<0.001**
Vegetarian	1392 (42.8%)	863 (37.2%)	529 (56.9%)	**<0.001**
BMI (kg/m^2^)	24.4 ± 3.4	24.7 ± 3.3	23.5 ± 3.5	**<0.001**
SBP (mmHg)	130.0 ± 20.0	131.6 ± 19.1	124.3 ± 20.4	**<0.001**
Glucose (mg/dL)	107.0 ± 22.4	107.4 ± 22.8	105.9 ± 21.3	0.096
Cre (mg/dL)	1.00 ± 0.31	1.07 ± 0.29	0.82 ± 0.29	**<0.001**
Hb (g/dL)	14.7 ± 1.6	15.3 ± 1.3	13.4 ± 1.3	**<0.001**
TG (mg/dL)	125.1 ± 75.1	130.1 ± 79.1	112.6 ± 62.5	**<0.001**
TCH (mg/dL)	180.9 ± 37.0	178.1 ± 36.8	187.8 ± 36.7	**<0.001**
LDL-C (mg/dL)	117.3 ± 31.8	116.6 ± 31.6	119.0 ± 32.2	0.052
HDL-C (mg/dL)	46.0 ± 13.8	43.4 ± 12.4	52.3 ± 14.9	**<0.001**
TG/HDL-C	3.2 ± 2.6	3.4 ± 2.8	2.5 ± 2.0	**<0.001**
TCH/HDL-C	4.2 ± 1.3	4.4 ± 1.3	3.8 ± 1.2	**<0.001**
LDL-C/HDL-C	2.8 ± 1.0	2.9 ± 1.0	2.5 ± 0.9	**<0.001**
L-spine BMD (g/cm^2^)	0.957 ± 0.156	0.988 ± 0.148	0.878 ± 0.146	**<0.001**
Right hip neck BMD (g/cm^2^)	0.710 ± 0.126	0.736 ± 0.122	0.647 ± 0.112	**<0.001**
Right hip total BMD (g/cm^2^)	0.820 ± 0.147	0.861 ± 0.133	0.720 ± 0.134	**<0.001**
Left hip neck BMD (g/cm^2^)	0.710 ± 0.126	0.735 ± 0.122	0.646 ± 0.112	**<0.001**
Left hip total BMD (g/cm^2^)	0.824 ± 0.149	0.865 ± 0.134	0.724 ± 0.136	**<0.001**

Values in bold represent significant values (*p* < 0.05). BMI: body mass index; SBP: systolic blood pressure; Cre: creatinine; Hb: hemoglobin; TG: triglyceride; TCH: total cholesterol; LDL-C: low-density lipoprotein cholesterol; HDL-C: high-density lipoprotein cholesterol.

**Table 2:** Simple linear regression for the data analysis: correlation between parameters and lipid profiles and atherogenic indexes by simple regression analysis.

	TG		TCH		LDL-C		HDL-C		TG/HDL-C		TCH/HDL-C		LDL-C/HDL-C	
	*r*	*p*	*r*	*p*	*r*	*p*	*r*	*p*	*r*	*p*	*r*	*p*	*r*	*p*
Age	*-0.066*	**<0.001**	*-0.048*	**0.006**	*-0.069*	**<0.001**	0.010	0.562	*-0.57*	**0.001**	*-0.045*	**0.010**	*-0.060*	**0.001**
Sex (male = 1, female = 0)	0.321	**<0.001**	0.318	**<0.001**	0.431	**<0.001**	0.323	**<0.001**	0.427	**<0.001**	0.321	**<0.001**	0.318	**<0.001**
BMI	0.270	**<0.001**	0.046	**0.008**	0.103	**<0.001**	*-0.353*	**<0.001**	0.293	**<0.001**	0.346	**<0.001**	0.322	**<0.001**
SBP	0.088	**<0.001**	0.026	0.139	0.031	0.077	*-0.102*	**<0.001**	0.092	**<0.001**	0.110	**<0.001**	0.096	**<0.001**
Glucose	0.177	**<0.001**	0.045	**0.010**	0.036	**0.042**	*-0.135*	**<0.001**	0.171	**<0.001**	0.150	**<0.001**	0.116	**<0.001**
Cre	0.104	**<0.001**	*-0.030*	0.082	*-0.011*	0.540	*-0.151*	**<0.001**	0.129	**<0.001**	0.129	**<0.001**	0.110	**<0.001**
Hb	0.182	**<0.001**	0.122	**<0.001**	0.171	**<0.001**	*-0.180*	**<0.001**	0.183	**<0.001**	0.245	**<0.001**	0.252	**<0.001**
Smoking	0.076	**<0.001**	0.115	**<0.001**	0.081	**<0.001**	0.110	**<0.001**	0.073	**<0.001**	0.076	**<0.001**	0.115	**<0.001**
Exercise	*-0.070*	**<0.001**	*-0.075*	**<0.001**	*-0.076*	**<0.001**	0.021	0.222	*-0.056*	**0.001**	*-0.074*	**<0.001**	*-0.076*	**<0.001**
Vegetarian	-0.024	0.167	*-0.190*	**<0.001**	*-0.169*	**<0.001**	*-0.075*	**<0.001**	0.001	0.935	*-0.067*	**<0.001**	*-0.074*	**<0.001**
L-spine BMD	0.100	**<0.001**	*-0.049*	**0.005**	*-0.009*	0.623	*-0.188*	**<0.001**	0.130	**<0.001**	0.136	**<0.001**	0.125	**<0.001**
Right neck BMD	0.121	**<0.001**	*-0.039*	**0.026**	0.003	0.882	*-0.185*	**<0.001**	0.149	**<0.001**	0.143	**<0.001**	0.132	**<0.001**
Right total BMD	0.145	**<0.001**	*-0.037*	**0.034**	*-0.003*	0.848	*-0.215*	**<0.001**	0.164	**<0.001**	0.163	**<0.001**	0.142	**<0.001**
Left neck BMD	0.112	**<0.001**	*-0.029*	0.101	0.015	0.407	*-0.178*	**<0.001**	0.138	**<0.001**	0.144	**<0.001**	0.137	**<0.001**
Left total BMD	0.144	**<0.001**	*-0.036*	**0.038**	*-0.002*	0.914	*-0.232*	**<0.001**	0.168	**<0.001**	0.178	**<0.001**	0.154	**<0.001**

Values in bold represent significant values (*p* < 0.05), and values in *italic* represent negative correlation. BMI: body mass index; SBP: systolic blood pressure; Cre: creatinine; Hb: hemoglobin, TG: triglyceride; TCH: total cholesterol; LDL-C: low-density lipoprotein cholesterol; HDL-C: high-density lipoprotein cholesterol.

**Table 3:** Simple linear regression for the data analysis: correlation between possible confounding parameters and BMDs by simple regression analysis.

	L-spine BMD		Right neck BMD		Right total BMD		Left neck BMD		Left total BMD	
	*r*	*p*	*r*	*p*	*r*	*p*	*r*	*p*	*r*	*p*
Age	*-0.118*	**<0.001**	*-0.335*	**<0.001**	*-0.151*	**<0.001**	*-0.328*	**<0.001**	*-0.131*	**<0.001**
Sex (male = 1, female = 0)	0.321	**<0.001**	0.318	**<0.001**	0.431	**<0.001**	0.323	**<0.001**	0.427	**<0.001**
BMI	0.327	**<0.001**	0.336	**<0.001**	0.380	**<0.001**	0.343	**<0.001**	0.395	**<0.001**
SBP	0.076	**<0.001**	0.024	0.166	0.134	**<0.001**	0.026	0.133	0.130	**<0.001**
Glucose	0.059	**0.001**	*-0.017*	0.342	0.037	**0.034**	*-0.004*	0.801	0.052	**0.003**
Cre	0.161	**<0.001**	0.105	**<0.001**	0.179	**<0.001**	0.117	**<0.001**	0.180	**<0.001**
Hb	0.183	**<0.001**	0.248	**<0.001**	0.303	**<0.001**	0.252	**<0.001**	0.291	**<0.001**
Smoking	0.076	**<0.001**	0.115	**<0.001**	0.081	**<0.001**	0.110	**<0.001**	0.073	**<0.001**
Exercise	0.022	0.214	*-0.013*	0.460	0.021	0.228	*-0.013*	0.462	0.035	**0.043**
Vegetarian	*-0.164*	**<0.001**	*-0.156*	**<0.001**	*-0.123*	**<0.001**	*-0.170*	**<0.001**	*-0.117*	**<0.001**

Values in bold represent significant values (*p* < 0.05), and values in italic represent negative correlation. BMI: body mass index; SBP: systolic blood pressure; Cre: creatinine; Hb: hemoglobin.

**Table 4:** Multiple linear regression for the data analysis: correlation between parameters of bone mineral density and atherogenic indexes by multiple regression analysis after adjustment.

	TG/HDL-C		TCH/HDL-C		LDL-C/HDL-C	
	*r*	*p*	*r*	*p*	*r*	*p*
Adjusted for age and sex						
L-spine BMD	0.080	**<0.001**	0.082	**<0.001**	0.068	**<0.001**
Right neck BMD	0.100	**<0.001**	0.089	**<0.001**	0.070	**<0.001**
Right total BMD	0.107	**<0.001**	0.087	**<0.001**	0.070	**<0.001**
Left neck BMD	0.085	**<0.001**	0.089	**<0.001**	0.076	**<0.001**
Left total BMD	0.114	**<0.001**	0.117	**<0.001**	0.086	**<0.001**
Adjusted for BMI						
L-spine BMD	0.038	**0.033**	0.026	0.137	0.022	0.219
Right neck BMD	0.057	**0.001**	0.030	0.083	0.027	0.123
Right total BMD	0.061	**0.001**	0.036	**0.040**	0.023	0.203
Left neck BMD	0.042	**0.018**	0.029	0.103	0.030	0.088
Left total BMD	0.063	**0.001**	0.049	**0.007**	0.032	0.079
Adjusted for age, sex, SBP, glucose, Cre, Hb, smoking, exercise, and vegetarian						
L-spine BMD	0.071	**<0.001**	0.070	**<0.001**	0.058	**0.001**
Right neck BMD	0.092	**<0.001**	0.077	**<0.001**	0.059	**0.002**
Right total BMD	0.088	**<0.001**	0.073	**<0.001**	0.047	**0.012**
Left neck BMD	0.076	**<0.001**	0.074	**<0.001**	0.062	**0.001**
Left total BMD	0.095	**<0.001**	0.095	**<0.001**	0.067	**<0.001**

Values in bold represent significant values (*p* < 0.05), and values in italic represent negative correlation. BMI: body mass index; SBP: systolic blood pressure; Cre: creatinine; Hb: hemoglobin; TG: triglyceride; TCH: total cholesterol; LDL-C: low-density lipoprotein cholesterol; HDL-C: high-density lipoprotein cholesterol.

**Table 5:** Multiple linear regression for the data analysis: correlation between parameters of bone mineral density and atherogenic index of TG/HDL-C in subgroups dividing by sex (female/male) and obesity (nonobese/obese) with multiple regression analysis after adjustment of age, SBP, glucose, Cre, Hb, smoking, exercise, and vegetarian.

Adjusted for age, SBP, glucose, Cre, Hb, smoking, exercise, and vegetarian	Female nonobese (656)		Female obese (273)		Male nonobese (1342)		Male obese (978)	
	*r*	*p*	*r*	*p*	*r*	*p*	*r*	*p*
L-spine BMD	0.076	0.076	0.029	0.663	0.061	**0.024**	0.004	0.907
Right neck BMD	0.134	**0.002**	0.113	0.087	0.060	**0.032**	*-0.012*	0.718
Right total BMD	0.089	**0.023**	0.166	**0.006**	0.070	**0.010**	*-0.049*	0.125
Left neck BMD	0.108	**0.011**	0.112	0.087	0.058	**0.039**	*-0.050*	0.134
Left total BMD	0.104	**0.008**	0.178	**0.003**	0.065	**0.016**	*-0.049*	0.118

Values in bold represent significant values (*p* < 0.05), and values in italic represent negative correlation. SBP: systolic blood pressure; Cre: creatinine; Hb: hemoglobin.

## Data Availability

The data used to support the findings of this study are available from the corresponding author upon request.
